# Supplementary material for: DNA G-Quadruplexes Contribute to CTCF Recruitment
Source: Int J Mol Sci. 2021 Jun 30;22(13):7090. doi: 10.3390/ijms22137090 (PMC8269367; doi:10.3390/ijms22137090)
Supplement: Supplementary file 1 [file ijms-22-07090-s001.zip › ijms-1269077-supplementary/supplementary_figs_and_tables.pdf]

DNA G-quadruplexes contribute to CTCF recruitment

P. Tikhonova, Iu. Pavlova, E. Isaakova, V. Tsvetkov*, A. Bogomazova, T. Vedekhina, A.V. Luzhin, R. Sultanov, V. Severov, K. Klimina, O.L. Kantidze, G. Pozmogova, M. Lagarkova*, A. Varizhuk*

SUPPORTING INFORMATION

Text box S1. Experimental details: bioinformatics....2

Table S1. ChIP-qPCR details: primer sequences....4

Table S2. Oligonucleotides tested for CTCF binding....4

Figure S1. CTCF complex with MYC-G4: detailed docking results....5

Figure S2. Relative distribution of G4-seq and HMG-protein peaks: summary of the whole-genome analysis....6

Table S3. HMG-protein and ATAC-seq datasets for the analyses of G4-seq and protein distributions in open chromatin....6

Table S4. HMG-protein and G4-seq peaks in open chromatin: Fisher test summary....6

Figure S3. HMG-protein and G4-seq peaks in open chromatin: Monte-Carlo simulations....7

Figure S4. Sequence logos of the motifs discovered in HMG protein-bound sites....8

Figure S5. Detailed analysis of enriched motifs in HMGB2-bound sites....9

Table S5. Sequences and secondary structures of HMGN3 motif-matching oligonucleotides....10

Figure S6. Secondary structures of HMGN3 motif-matching oligonucleotides: verification by optical and electrophoretic methods....10

Table S6. Sequences and secondary structures of HMGB2 motif-matching oligonucleotides....11

Figure S7. Secondary structures of HMGB3 motif-matching oligonucleotides: verification by optical and electrophoretic methods....11

Table S7. Sequences and secondary structures of HMGN1 motif-matching oligonucleotides....12

Figure S8. Secondary structures of HMGN1 motif-matching oligonucleotides: verification by optical and electrophoretic methods....12

Text box S1. Experimental details: bioinformatics

1. Relative distribution of G4 motifs, i-motifs, CpG islands and CTCF occupancy sites
G4 motif mining was performed using the updated version of imGQFinder (1) with the following settings: number of G-tetrads ≥ 3, maximum loop length:7; maximum number of defects (mismatches, vacancies or bulges of 1-3 nt): 1. The source code of imGQFinder version 2.2.0 is available at https://github.com/RCPCM-GCB/ImGQFinder. It is implemented in Python 3 under the MIT license. To select the motifs that are likely folded in the human erythromyeloblastoid leukemia (K562) cell line, we intersected the whole G4 motif set with BG4 peaks (GSE107690), and discarded the motifs that are localized outside high-confidence BG4 peaks. I-Motif (iM)-mining was performed using G4-iM Grinder (2), algorithm M3. The minimal C-tract length was set to 5 nt, based on the previously reported sequence criteria for iM stability under physiological conditions (3), and the maximum loop length was set to 7 nt. For the functional profiling of the iM-harboring genes, we used the g:Profiler toolset (4). Coordinates of the CGIs were downloaded from UC Santa Cruz University (USCU) database, and CTCF ChIP-seq datasets were downloaded from ENCODE (ENCSR000AKO and ENCSR822CEA for K562 and neural cells, respectively).

2. Relative distribution of CpG islands and BG4, DNMT1, and CTCF occupancy sites
Data sets for G4-ChIP-seq peaks (high confidence BG4 peaks) in K562 cells were downloaded from Gene Expression Omnibus (GSE107690). DNMT1 and CTCF ChIP-seq data sets were downloaded from ENCODE (ENCSR987PBI and ENCSR000AKO, respectively). Coordinates of the CpG islands (CGIs) were downloaded from the USCU database. The subsets of BG4/DNMT/CTCF-overlapping CGIs and CGI/DNMT/CTCF-overlapping BG4 peaks were obtained using Bedtools (5) and Galaxy Version 1.0.6 intersect tools (localization in the plus or minus strand was not taken into account). For sampling analysis of G4/CGI/DNMT localization relative to chromatin loop boundaries, the K562 5C dataset from ENCODE ENCODE/Dekker Univ. Mass. was used, and the data were visualized with the Integrative genomics viewer (Igv).

3. Methylation levels in CpG islands, BG4 and CTCF occupancy sites
Methylation levels at CpG sites obtained from whole-genome shotgun bisulfite sequencing (WGBS) assay (ENCODE, ENCSR765JPC) were downloaded from Gene Expression Omnibus (GSM2308597; ENCLB742NWU). CpG sites with less than 5x coverage (14% of 58607924 sites) were discarded, and the remaining sites were ported to the human genome release GRCh37 (hg19) using the batch coordinate converter (liftover) tool of Galaxy Version 1.0.6 (approximately 93% sites mapped to hg19). Average CpG methylation levels in CGIs, BG4 peaks, and CTCF peaks were calculated using Galaxy General text tools. Briefly, CpG sites with high-confidence methylation levels were intersected with indexed CGIs, BG4 peaks or CTCF peaks, then grouped based on the indexes, and the average methylation values for each index were computed. Average methylation levels in CGI-BG4-CTCF intersections were computed analogously, except that indexed overlapping pieces of 'CGI'/ 'BG4'/ 'CTCF' genomic intervals were used instead of the initial intervals.

4. G4 association with HMG proteins: whole-genome analysis
The G4-seq dataset (6) was downloaded from Gene Expression Omnibus (GSM3003539). ChIP-seq dataset for HMGN3 in chronic myeloid leukemia cells K652 cell line was downloaded from ENCODE (ENCSR000DOB). ChIP-seq datasets for HMGB2 in fetal lung fibroblasts IMR-90 p10/IMR-90 p28 cells and HMGN1 in CD4+ T-cells were downloaded from Gene Expression Omnibus (GSM2589818/GSM2589819 and GSM630809, respectively). For the whole-genome analysis of G4-protein colocalization, we used the downloaded datasets without additional processing or filtering, except that the HMGN1 peak coordinates were ported to the human genome release GRCh37 (hg19) using the batch coordinate converter (liftover) tool of Galaxy Version 1.0.6.

G4-seq peaks were intersected with protein ChIP-seq peaks, and the results were used to estimate G4-seq coverage (average per 100000 bp) inside protein peaks as compared with the whole-genome average G4-seq coverage, which was calculated based on the initial G4-seq data dataset (6). To evaluate the significance of the G4-protein correlations, Monte-Carlo simulations were performed. We randomized protein peaks 1000 times over the genome and each time measured the intersection with G4-seq peaks. An empirical p-value was calculated by comparing the number of true G4-protein intersections with these 1000 randomized intersections. All intersections and randomizations were performed using Bedtools (5).

5. G4 association with HMG proteins: open-chromatin analysis
For the open-chromatin analysis, we remapped HMG protein ChIP-seq peaks on hg19 to exclude possible artifacts resulting from variations in the previously used peak-calling workflows. We also verified whether the difference in peak coverage and sample size affects the analysis. We applied four different filters to the protein datasets and assessed the G4-protein correlation for each filtered dataset. Filter 1: the ChiP-seq score threshold for high-confidence peaks was set to 150; filter 2: the ChiP-seq score threshold was set to 600; filter 3: for each protein, a specific 'optimal' ChIP-seq threshold was chosen so that 1.5% peaks were selected, and others were discarded; filter 4: 100 top-scoring peaks were selected, and others were discarded. G4-seq and filtered protein ChIP-seq peaks were then intersected with open

chromatin sites revealed by assay for transposase-accessible chromatin using sequencing (ATAC-seq) data for respective cell lines.
The ATAC-seq datasets for IMR-90 and CD4+ T-cells were downloaded from Gene Expression Omnibus (dataset GSM1418975 and merged datasets GSM3258583 and GSM3258584, respectively). We started from raw data and performed peak calling following a previously developed pipeline [https://github.com/ay-lab/ATACProc]. Raw data (medium-depth) were downloaded from the NCBI SRA Run Selector. For each run, the ATAC-seq peaks were called using the MACS2 algorithm with default settings, and the resulting datasets were merged for each cell line. Protein ChIP-seq peaks within ATCA-seq peaks in the respective cell line and G4-seq peaks within ATAC-seq peaks were intersected, and the significance of the G4-protein correlation was verified using the Fisher test. Monte-Carlo simulations were performed as described in the previous subsection.

6. Relative distribution of G4-harboring HMG-protein-bound sites and CTCF sites

Whole-genome HMG-protein ChIP-seq peaks and their subsets containing G4-seq-harboring peaks were intersected with CTCF occupancy sites in respective cell lines using Bedtools (21). CTCF ChIP-seq datasets for proliferating IMR90 and K562 cells were downloaded from ENCODE (ENCSR000EFI, and ENCSR000AKO, respectively). CTCF ChIP-seq data for CD4+ T cells were downloaded from Gene Expression Omnibus (GSM1936635).

References

1. Varizhuk, A., Ischenko, D., Tsvetkov, V., Novikov, R., Kulemin, N., Kaluzhny, D., Vlasenok, M., Naumov, V., Smirnov, I. and Pozmogova, G. (2017) The expanding repertoire of G4 DNA structures. *Biochimie*, **135**, 54-62.
2. Belmonte-Reche, E. and Morales, J.C. (2019) G4-iM Grinder: when size and frequency matter. G-Quadruplex, i-Motif and higher order structure search and analysis tool. *NAR Genomics and Bioinformatics*, **2**.
3. Wright, E.P., Huppert, J.L. and Waller, Z.A.E. (2017) Identification of multiple genomic DNA sequences which form i-motif structures at neutral pH (vol 45, pg 2951, 2017). *Nucleic Acids Research*, **45**.
4. Reimand, J., Kull, M., Peterson, H., Hansen, J. and Vilo, J. (2007) g : Profiler - a web-based toolset for functional profiling of gene lists from large-scale experiments. *Nucleic Acids Research*, **35**, W193-W200.
5. Quinlan, A.R. and Hall, I.M. (2010) BEDTools: a flexible suite of utilities for comparing genomic features. *Bioinformatics*, **26**, 841-842.
6. Marsico, G., Chambers, V.S., Sahakyan, A.B., McCauley, P., Boutell, J.M., Di Antonio, M. and Balasubramanian, S. (2019) Whole genome experimental maps of DNA G-quadruplexes in multiple species. *Nucleic Acids Research*, **47**, 3862-3874.

Table S1. ChIP-qPCR details: primer sequences

code	sequence	location
USP24_forward<br>USP24_reverse	CCACTGGCTGTCCCTAGATCA<br>TCCTACCTGCACTACCATGC	Chr 1, USP24 intron 21
STAT3_forward<br>STAT3_reverse	TGAGTGAAACAGGGAGTCAAG<br>AGAAGGACATCAGCGGTAAG	Chr 17, STAT3 intron and exon 20
VEGFA_forward<br>VEGFA_reverse	AGCCCATTCCCTCTTTAGCC<br>ACACACTCACTCACCCACAC	Chr 6, VEGFA promoter
MYC_forward<br>MYC_reverse	CTCAGAGGCTTGGCGGGAAAA<br>CAGCGAGTTAGATAAAGCCCCG	Ch8, MYC promoter
VGPS4A_forward<br>VGPS4A_reverse	GGCAACCACTGCTAATCTACTT<br>GCCACAAAGGACCACCTATT	VPS4A intron 1

Table S2. Oligonucleotides tested for CTCF binding

code	sequence	gene
MYC-G4	TGGGGAGGGTGGGGAGGGTGGGGAAGG	MYC
MYC-iM	TCCCCACCTTCCCCACCCTCCCCACCCTCCCCA	
BDN-G4	GGGGGGCGGGGGGCGGGGGGGGGGG	BDNF
BDN-iM	CCCCCCCCCCCGCCCCCCGCCCCCC	
SHA-G4	GGGGGTGGGGGGTGGGGGGAGGGGGG	SHANK1
SHA-iM	CCCCCCTCCCCCCACCCCCCACCCCC	

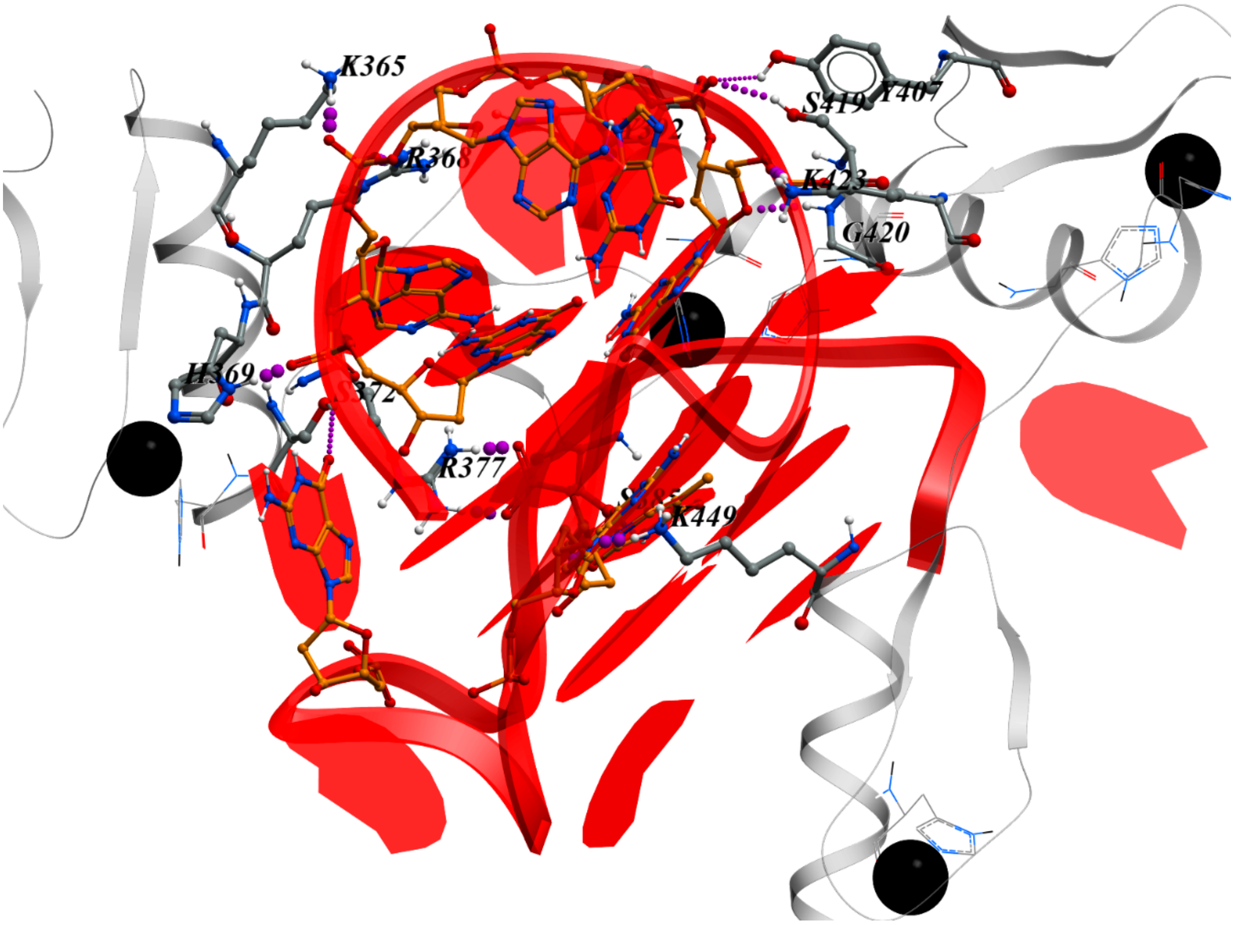

Figure S1. CTCF complex with MYC-G4: detailed docking results. The best binding energy conformation of the MYC-G4 (red) complex with CTCF (grey). Amino acid residues and nucleotides that form hydrogen bonds are highlighted by rendering. Histidine residues that form coordination bonds with zinc ions (black) are also marked. Atom colouring: MYC-G4 carbon – copper, protein carbon – grey, oxygen – red, nitrogen – blue, and hydrogen – white (hydrogens are only shown in the polar groups). Hydrogen bonds are shown as dotted lines; the thickness illustrates the binding energy.

In the best binding energy conformation, non-ester oxygens of the negatively charged G4 backbone phosphates form strong hydrogen and ionic bonds with the positively charged amino acid groups of CTCF, i.e., the guanidino groups of Arg377 and Arg368, protonated amino groups of Lys365, Lys449, and Lys423, and His369. H-bonds are also formed between backbone phosphates and hydroxyl groups of Tyr392, Tyr407, and Ser419 residues. In addition to that, two nucleotide heterocyclic residues participate in H-bonding with the protein. The imido NH group of the thymine residue from the MYC-G4 propeller loop that joins guanosines G6 and G8 is a donor for the hydroxyl oxygen of Ser385 residue, and the backbone NH group of Gly420 is a donor for the ribose oxygen of the guanosine residue G19.

A

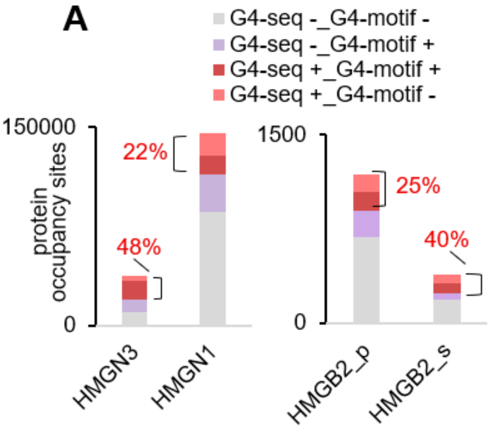

B

	G4-seq ∩ protein (real:predicted by chance)	X^2 (1, N = 100000)
HMGN3	123:11	1167
HMGB2	12:0.8	160
HMGN1	155:59	155

Figure S2. Relative distribution of G4-seq and HMG-protein peaks: summary of the whole-genome analysis. (**A**) Bar graphs illustrating the numbers and portions of G4-seq/G4-motif-overlapping and non-overlapping protein peaks in K562 (HMGN3), proliferating IMR90 (HMGB2_p) and senescent (HMGB2_s) and CD4+ T cells (HMGN1). (**B**) Summary of Chi-square statistics for G4-seq-protein intersections. G4-seq ∩ protein is the average coverage of G4-protein intersections per 100000 bp.

Table S3. HMG-protein and ATAC-seq datasets for the analyses of G4-seq and protein distributions in open chromatin.

cell line, protein		K562, HMGN3	IMR-90, HMGB2	CD4+ T-cells, HMGN1
total number/median length of ATAC-seq peaks		19109/ 601	55700/ 1451	27488/ 495
total number/ median length (bp) of protein peaks	optimal threshold	233/ 496	595/ 467	93/ 245
	top-100	100/ 496	100/ 467	100/245
	threshold 150	10552 /694	290/ 467	347/245
	threshold 600	229/265	102/694	3/451

Table S4. HMG-protein and G4-seq peaks in open chromatin: Fisher test summary. The numbers of intersecting and non-intersecting G4/protein peaks within ATAC-seq peaks are given. P-value was < 0.00001 in all cases.

		K562, open chromatin		IMR-90, open chromatin		CD4+ T-cells, open chromatin	
		HMGN3	G4-seq	HMGB2	G4-seq	HMGN1	G4-seq
protein filter: 'opt. threshold'	intersect.	95	227	171	450	25	45
	non-inters.	112	13951	394	39483	51	16284
protein filter: 'top 100'	intersect.	47	108	36	76	36	61
	non-inters.	53	14070	64	39857	64	16268
protein filter: 'threshold 150'	intersect.	3642	8982	95	232	96	172
	non-inters.	4366	5196	177	39701	193	16157
protein filter: 'threshold 600'	intersect.	93	223	36	76	0	0
	non-inters.	111	13955	64	39857	3	16329

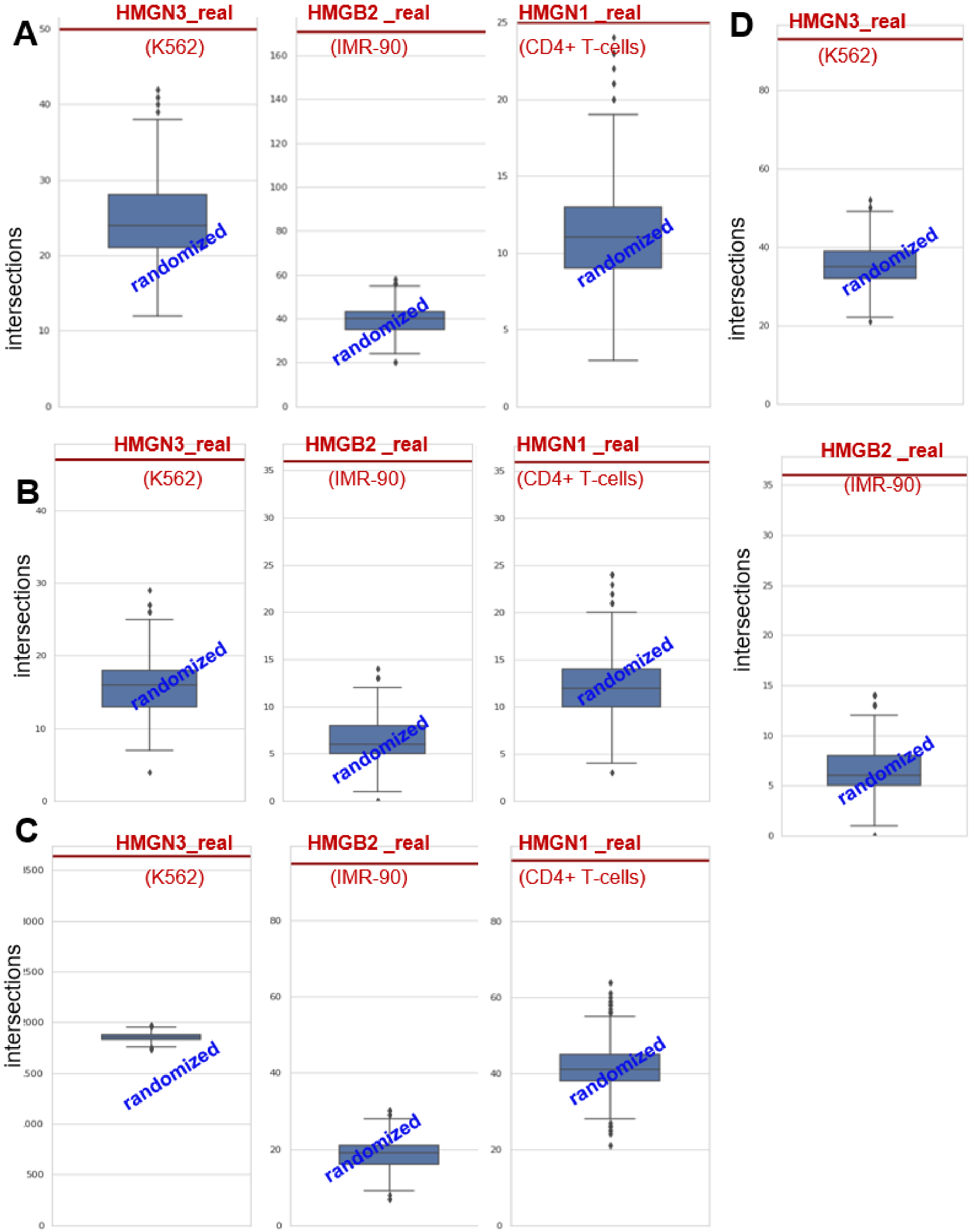

Figure S3. HMG-protein and G4-seq peaks in open chromatin: Monte-Carlo simulations. Red lines indicate true number of G4-seq-overlapping protein peaks within ATAC-seq peaks for the respective cell line. (**A**) Protein filter: optimal threshold (1.5% of all ChIP-seq peaks). (**B**) Protein filter: top 100. (**C**) Protein filter: threshold 150. (**D**) Protein filter: threshold 600 (for HMGN1, the number of peaks was insufficient for proper analysis)**.**

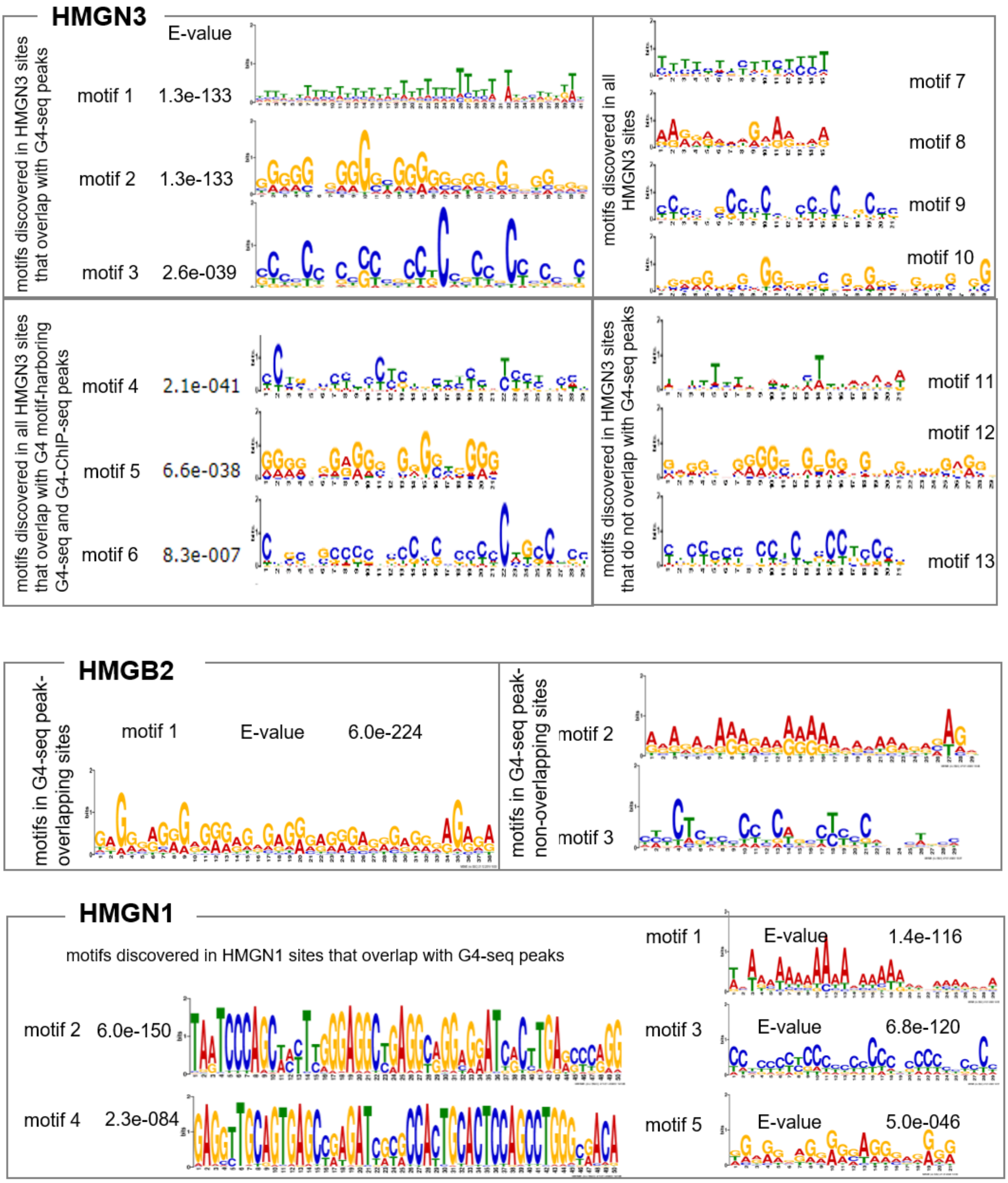

Figure S4. Sequence logos of the motifs discovered in HMG protein-bound sites.

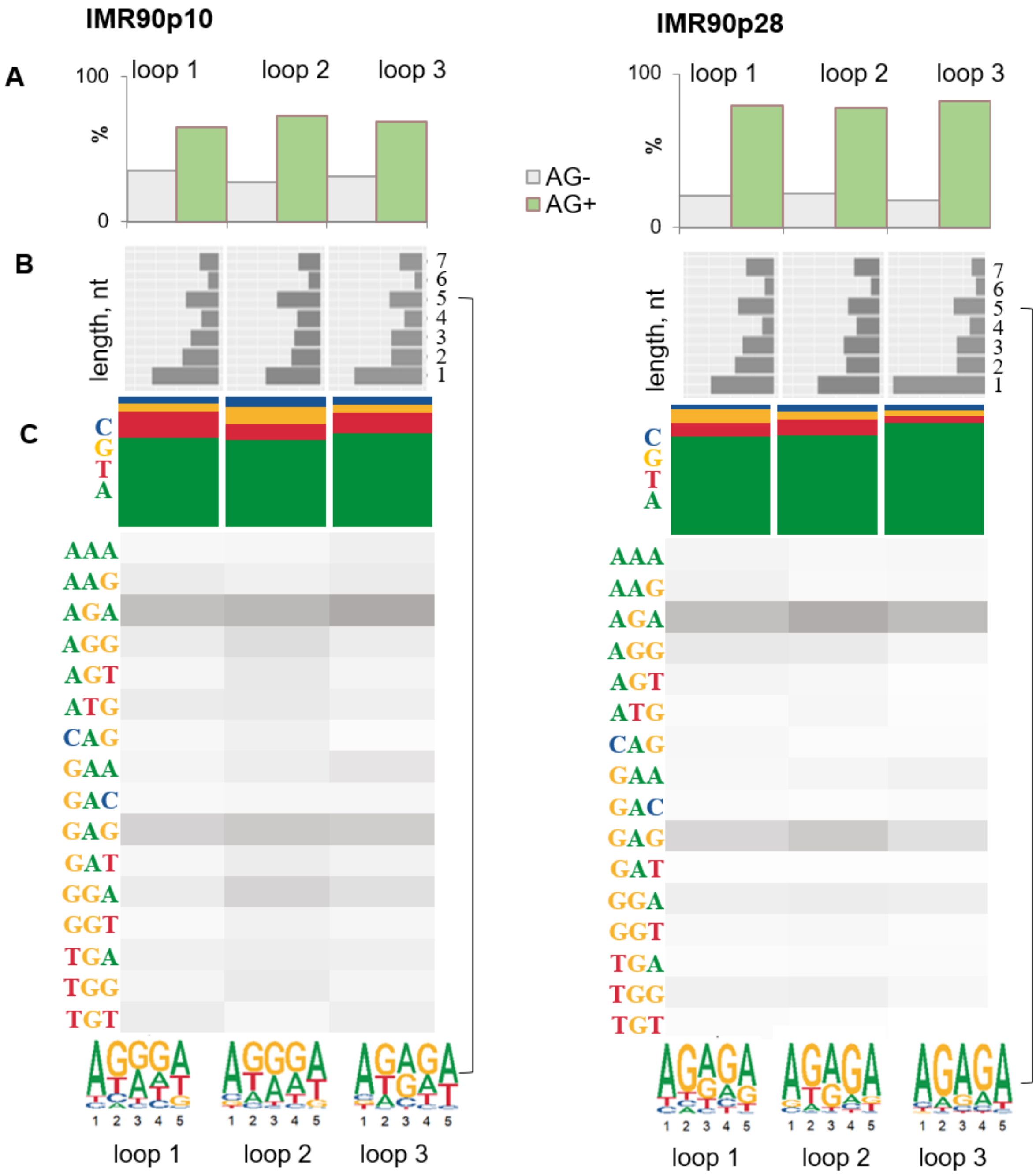

Figure S5. Detailed analysis of enriched motifs in HMGB2-bound sites. A: General AG frequency in G4 loops. B: Loop length distribution. C: Relative frequencies of nucleotides/trinucleotides in G4 loops and sequence logos for 5-nt loops.

Table S5. Sequences and secondary structures of HMGN3 motif-matching oligonucleotides

code	description	sequence, 5'-3'	secondary structure**
N3-1/ N3C-1	HMGN3 motif 7_best match/ complement	TTTTCTTCTTCTTTT AAAAGAAGAAGAAAA	duplex
N3-2/ N3C-2	HMGN3 motif 8_best match /complement	AAGGAAAAGAAAAAA TTTTTTCTTTTCCTT	duplex
N3-M1	HMGN3 motif 1_ matched sequences*	GGGGAGCGGGGCGGGCTAGGG	pG4
N3-M2		GGAGTAGGGGCAGGGGCAGGG	pG4
N3-M3		GAGGCGGGGAGGGGGCTTGGG	pG4, mostly intermolecular
N3-M4	HMGN3 motif 10_ matched sequences*	GGGCACGCAGGCGGCTGCGGGGGCGGAGG	unidentified, intermolecular
N3-M5		GGAGCAGGTGGGGAGCAGGCCGGAAGAGG	aG4
N3-M6		GGGGCCAGTGGACGCGGGGAGCCTGCGGG	mG4
N3-M7		GGGGACGAAGGGGGCGCGGTGCCTCCTGG	mG4
hair	Additional sequences for selectivity analysis	CAATCGGATCGAATTCGATCCGATTG	hairpin
A20		AAAAAAAAAAAAAAAAAAAA	-
T20		TTTTTTTTTTTTTTTTTTTT	-
CT1	Positive control	GGTGACAGGGGTATGGGGAGGGG	pG4

*G4-prone sequences from protein peaks that match specified motifs with p-value < 0.001
** Based on the data in Fig. S6. Abbreviations: pG4, parallel G-quadruplex; aG4, antiparallel quadruplex, mG4, mixed-type quadruplex.

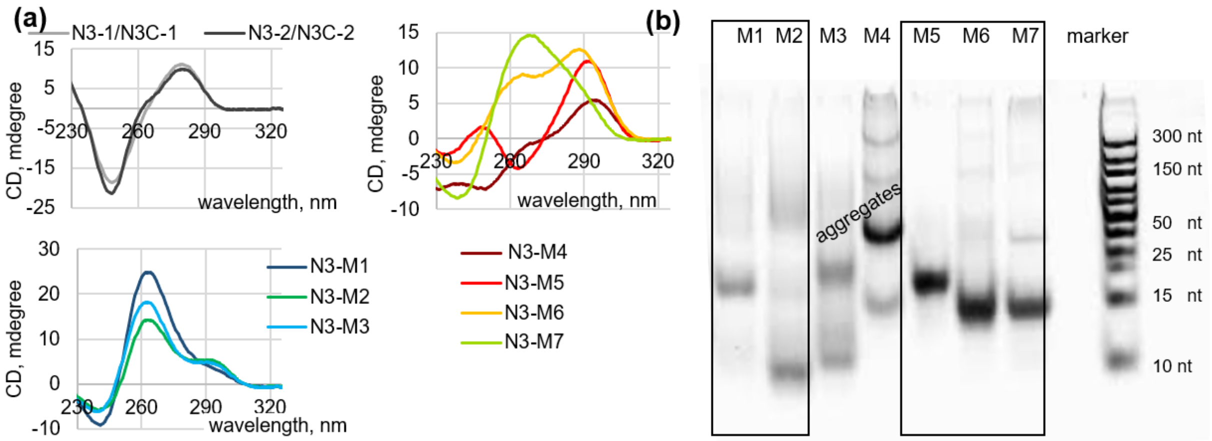

Figure S6. Secondary structures of HMGN3 motif-matching oligonucleotides: verification by optical and electrophoretic methods. (**a**) Circular dichroism (CD) spectra. Conditions: 3μM ODN in 140 mM potassium-phosphate buffer, pH 7.2, supplemented with 20 mM NaCl. Temperature: 20 °C. (**b**) Nondenaturing PAGE of the G4s M0-M7. Conditions: 5 μM ODN; 20% gel, standard TBE buffer supplemented with 10 mM KCl; staining with SYBR Gold. All ODNs were preannealed at a concentration of 40 μM (conditions similar to those used in microscale thermophoresis assays).

Table S6. Sequences and secondary structures of HMGB2 motif-matching oligonucleotides

code	description	sequence, 5'-3'	secondary structure**
B2-1/<br>B2C-1	HMGB2 motif 2_best match/<br>complement	AGAGAGAGAGAAAGAAAGAAAGAGAGAGA<br>TCTCTCTCTTTCTTTCTTTCTCTCTCTCT	duplex
B2-M0	HMGB2 motif 1_ matched sequence*	GGGAGGGAGGGAGGG	pG4
B2-M1		GGGAGGGGAGGGGAGGG	pG4
B2-M2		GGGAGGGAGGGGAGGGG	pG4
B2-M3		GGGAGGGAGGAAGGGAGGG	pG4
B2-M4		GGGAGGGAGAGAGGGAGGG	pG4
B2-M5		GGGCTGGGGGGGGCGGG	pG4
B2-M6		GGGAGAGGGAGAGGGAGAGGG	mG4
B2-M7		GGGCCCTGGGAACGGGGAGGG	mG4
hair	Additional sequences for selectivity analysis	CAATCGGATCGAATTCGATCCGATTG	hairpin
A20		AAAAAAAAAAAAAAAAAAAA	-
T20		TTTTTTTTTTTTTTTTTTTT	-
22CTA		AGGGCTAGGGCTAGGGCTAGGG	aG4
HRAS		TCGGGTTGCGGGCGCAGGGCACGGGCG	aG4
CT1	Positive control	GGTGACAGGGGTATGGGGAGGGG	pG4

* G4-prone sequences from protein peaks that match the specified motif with p-value < 0.1 and bear characteristic loop features shown in Fig. S5.
** Based on the data in Fig. S7

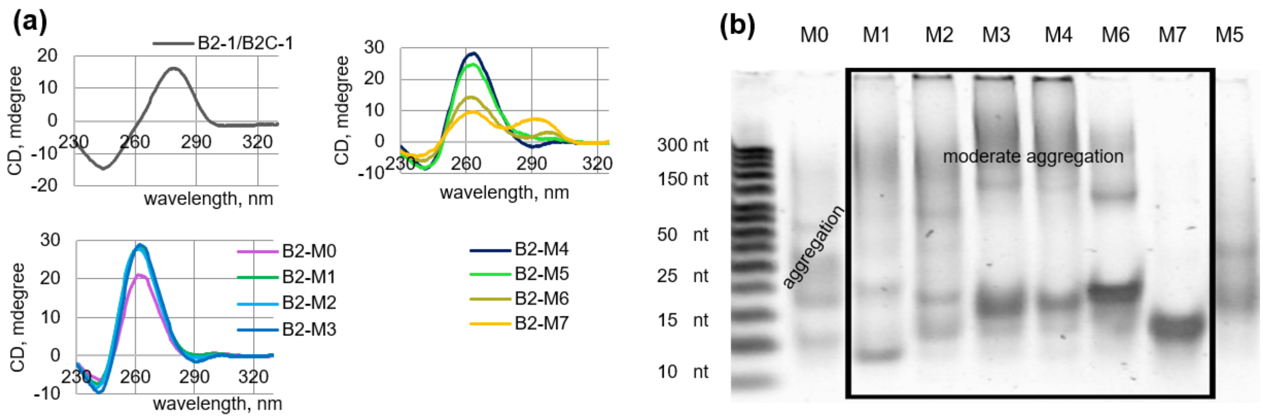

Figure S7. Secondary structures of HMGB3 motif-matching oligonucleotides: verification by optical and electrophoretic methods. (**a**) Circular dichroism (CD) spectra. Conditions: 3μM ODN in 140 mM potassium-phosphate buffer, pH 7.2, 20 mM NaCl. Temperature: 20 °C. (**b**) Nondenaturing PAGE of the G4s M0-M7. Conditions: 5 μM ODN; 20% gel; standard TBE buffer supplemented with 10 mM KCl; staining with SYBR Gold. All ODNs were preannealed at a concentration of 40 μM (conditions similar to those used in microscale thermophoresis assays).

Table S7. Sequences and secondary structures of HMGN1 motif-matching oligonucleotides

code	description	sequence, 5'-3'	secondary structure**
N1-1/<br>N1C-1	HMGN1 motif 1 (5'-truncated)/<br>complement	AAAAAAAAAAAAAAAAAAAA<br>TTTTTTTTTTTTTTTTTTTT	duplex
N1-2/<br><br>N1C-2	HMGN1 motif 2_best match/<br><br>complement	TAATCCCAGCTATTTGGGAGGCTGAGGCAGG AGGATCACTTGAGCCCAGG /<br>CCTGGGCTCAAGTGATCCTCCTGCCTCAGCC TCCCAAATAGCTGGGATTA	duplex
N1sh-2/<br>N1shC2	HMGN1 motif 2 (5'-truncated)/<br>complement	GGGAGGCTGAGGCAGG<br>CCTGCCTCAGCCTCCC	duplex or mG4
N1-3/<br>N1C-3	HMGN1 motif 3_best match/<br>complement	CCTCCCCTCCCTCCCCCCTCCCCCTCCCC<br>GGGGAGGGGGAGGGGGGAGGGAGGGGAGG	iM + pG4 (partly intermolecular)
N1sh-3<br>N1shC3	HMGN1 motif 3 (3'-truncated)/<br>complement	CCCTCCCTCCCTCCC<br>GGGAGGGAGGGAGGG	ssDNA + intermolecular pG4
N1-5<br>N1C-5	HMGN1 motif 5_best match/<br>complement	GGGGAGGGGAGGGGAGAAGAGGGG<br>CCCCTCTTCTCCCCTCCCCTCCCC	duplex or pG4
Tel26	Additional sequences for selectivity analysis	AAAGGGTTAGGGTTAGGGTTAGGGAA	mG4
22CTA		AGGGCTAGGGCTAGGGCTAGGG	aG4
htel21T18		GGGTTAGGGTTAGGGTTTGGG	aG4
hair		CAATCGGATCGAATTCGATCCGATTG	hairpin
A20		AAAAAAAAAAAAAAAAAAAA	-
T20		TTTTTTTTTTTTTTTTTTTT	-
CT1	Positive control	GGTGACAGGGGTATGGGGAGGGG	pG4

* G4-prone sequences from protein peaks that match the specified motif with p-value < 0.1 and bear characteristic loop features shown in Fig. S5.
** Based on the data in Fig. S8

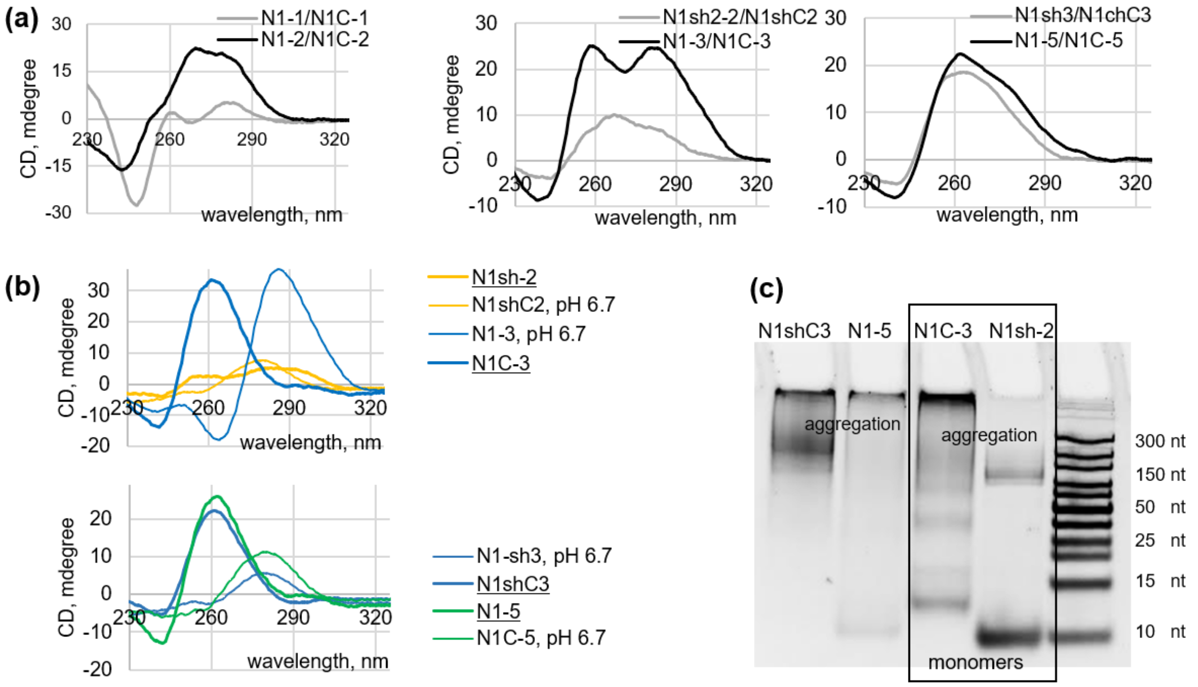

Figure S8. Secondary structures of HMGN1 motif-matching oligonucleotides: verification by optical and electrophoretic methods. (**a**) Circular dichroism (CD) spectra of presumed duplexes. (**b**) CD spectra of G/C-rich ODNs (individual strands). Conditions: 3μM ODN in 140 mM potassium-phosphate buffer, pH 7.2, 20 mM NaCl. Temperature: 20 °C. (**c**) Nondenaturing PAGE of the G4s M0-M7. Conditions: 5 μM ODN; 20% gel; standard TBE buffer supplemented with 10 mM KCl; staining with SYBR Gold. All ODNs were preannealed at a concentration of 40 μM (conditions similar to those used in microscale thermophoresis assays).
